# Underreporting of conflicts of interest in clinical practice guidelines: cross sectional study

**DOI:** 10.1186/1472-6939-14-19

**Published:** 2013-05-03

**Authors:** Julie Bolette Brix Bindslev, Jeppe Schroll, Peter C Gøtzsche, Andreas Lundh

**Affiliations:** 1 The Nordic Cochrane Centre, Rigshospitalet Department 7811, Blegdamsvej 9, Copenhagen Ø, DK-2100, Denmark; 2 Faculty of Health and Medical Sciences, University of Copenhagen, Copenhagen, Denmark

**Keywords:** Clinical practice guidelines, Conflicts of interest, Disclosure, Transparency

## Abstract

**Background:**

Conflicts of interest affect recommendations in clinical guidelines and disclosure of such conflicts is important. However, not all conflicts of interest are disclosed. Using a public available disclosure list we determined the prevalence and underreporting of conflicts of interest among authors of clinical guidelines on drug treatments.

**Methods:**

We included up to five guidelines published from July 2010 to March 2012 from each Danish clinical specialty society. Using the disclosure list of the Danish Health and Medicines Authority, we identified author conflicts of interest and compared them with the disclosures in the guidelines. For each guideline we extracted methodological characteristics of guideline development.

**Results:**

Forty-five guidelines from 14 specialty societies were included. Of 254 authors, 135 (53%) had conflicts of interest, corresponding to 43 of the 45 guidelines (96%) having one or more authors with a conflict of interest. Only one of the 45 guidelines (2%) disclosed author conflicts of interest. The most common type of conflict of interest (83 of the 135) was being a consultant, an advisory board member or a company employee. Only 10 guidelines (22%) described the methods used for guideline development, 27 (60%) used references in the text and 11 (24%) graded the types of evidence.

**Conclusions:**

Conflicts of interest were common, but disclosures were very rare. Most guidelines did not describe how they were developed and many did not describe the evidence behind specific recommendations. Publicly available disclosure lists may assist guideline issuing bodies in ensuring that all conflicts are disclosed.

## Background

The amount of medical information is overwhelming and it increases rapidly [1]. Clinical practice guidelines are therefore an important tool for assisting clinicians and patients in clinical decision-making [2]. Clinical practice guidelines should be based on valid scientific evidence, critical assessment of that evidence, and objective clinical judgement that relates the evidence to the needs of practitioners and patients [3]. However, treatment recommendations in guidelines are often based on expert opinion and low levels of evidence, which make them prone to biases and prejudices [4]. Conflicts of interest among guideline authors may therefore pose problems [4], as they may influence treatment recommendations [5,6].

The potential effects of conflicts of interest might have profound effects on health care because guidelines are written to influence the practice of physicians [7] and can be used for economic prioritisation [8]. Studies of conflicts of interest have found that up to 87% of guideline authors had interactions with drug companies [8,9]. Many of these conflicts are not disclosed because guideline issuing bodies do not publish this information [10] or because the authors choose not to disclose them [11]. This makes it likely that previous studies, which have relied on disclosed information, have underestimated the actual prevalence of conflicts of interest.

In Denmark, a nation of approximately 5.6 million inhabitants, there are around 22,500 practising physicians, 8.5% of which have a registered affiliation with a drug company [12]. Any physician wishing to receive payment from a drug company is obliged to apply for permission through the Danish Health and Medicines Authority and all physicians with permissions are named on a publicly available list with information on company and type of affiliation, but without financial data [13]. Similar to the US Physician Payments Sunshine Act [14], the list makes it possible to study the level of underreporting of conflicts of interest among guideline authors [15].

Our aims were:

•to determine the prevalence and types of conflicts of interest among authors of clinical practice guidelines published by Danish specialty societies;

•to estimate the proportion of disclosed conflicts of interest;

•to describe the methodology used in the guidelines.

## Methods

In Denmark there are 38 Danish specialty societies as defined by the list of the Organization of Danish Medical Societies [16]. Currently, national clinical practice guidelines are produced by individual societies and some are produced jointly. Each society differs in relation to how many guidelines they produce and how frequent they are updated. While the guidelines are not officially sanctioned by national or local health authorities, nor directly affect coverage decisions, the guidelines set standards for best practice and are used by physicians for clinical decision making. We sampled guidelines from each of the 38 Danish specialty societies, but excluded guidelines from non-clinical societies (e.g. radiology and pathology).

### Selection of guidelines

In March 2012, using the website for each specialty society, one observer included the five most recent drug guidelines published from July 2010. We limited the number to five in order to avoid clustering by specialties with many guidelines. As we focused on conflicts of interest in relation to drugs, we selected guidelines with a focus on drug treatment of medical conditions. For example, for anaesthesia we included a guideline on strategies for sedation, but excluded one on tracheotomy. In case of multiple guidelines of similar publication date, we selected them randomly. Societies without guidelines on their website were contacted by e-mail to determine whether any guidelines had been published. Guidelines referenced on the societies' website that had been developed by other national or international organisations were not considered a guideline for the particular society. Guidelines made in collaboration between different specialties were included in a separate category. Some societies had not produced five guidelines meeting our selection criteria and in those cases we included those that were available. A second observer verified the selection of guidelines according to our criteria. For each society, we obtained information as to whether the society had produced an instruction manual for guideline preparation.

### Guideline information

For each included guideline, two observers independently extracted information on title, date, number of authors, names of authors, funding of guideline and disclosures of conflicts of interest into a standardised datasheet. Disagreements were resolved through discussion. We contacted the specialty society for missing information on date of publication and funding.

### Conflicts of interest

We used the publicly available Danish registry of authorization to practise medicine to ensure the identity of authors and that they were physicians [17]. For each author, information on conflicts of interest was identified using the disclosure list of the Danish Health and Medicines Authority [13]. The list is updated continuously and we used three different versions from the period June 2010 to March 2012. If we were uncertain about whether a guideline author matched a physician on the disclosure list (e.g. due to variation in spelling of the name), we contacted the guideline issuing specialty society and the Danish Health and Medicine Authority for clarification. Two observers extracted information on conflicts of interest and disagreements were resolved through discussion. We coded a conflict of interest to be present if an author had an affiliation with a drug company up to 3 years prior to the published guideline, similar to the ICMJE criteria for biomedical journals [18]. When we were in doubt, we obtained additional information by applying for this through the Danish Health and Medicines Authority.

The type of conflict of interest with drug companies was classified into the following categories, which we defined a priori based on our previous experience with the disclosure list [12]:

•Consultant/advisory board member/employee

•Speaker/educational activities

•Investigator/research collaboration

•Equity/stockholder

Authors who have received reimbursement for conference expenses or fees for single activities such as speaking at only one meeting are not listed on the disclosure list.

For societies with an instruction manual for guideline preparation, we coded whether the manual contained information on disclosure of conflicts of interest.

### Pilot

Our datasheets were developed using a pilot version on one guideline on drug treatments from each of five randomly selected specialty societies.

### Guideline methodology

We initially planned to use the AGREE II instrument to assess the reporting of guideline methodology [19], but due to the low standards of reporting we encountered in our pilot study, we decided to use a simplified version adapted for key domains. For each guideline, two observers independently extracted information on description of methods for guideline development, use of references, and grading of types of evidence. Use of references was categorized as: references in text (for example when a particular drug was recommended in the text and a trial of this drug was cited), references, but not in text (when the references were at the end of the guideline only), and no references. We coded grading of evidence to be present if authors described the levels of evidence behind specific recommendations or the strength of recommendations according to a system (for example Ia, Ib, IIa, IIb, III, IV or A, B, C, D).

### Data analysis

We calculated the prevalence of disclosed and undisclosed conflicts of interest overall, at guideline level, and at specialty society level. For authors with conflicts of interest, we calculated the proportions of the individual types of conflicts of interest. Guidelines made in collaboration between different specialties were analysed separately.

### Sensitivity analysis

The estimated overall prevalence of authors with conflicts of interest depends on the number of authors per society and the prevalence of conflicts of interest at society level. We therefore performed a simple sensitivity analysis to test the robustness of our results. We estimated the prevalence as an average of the mean prevalence of conflicts of interest among individual societies, assigning each society the same weight.

We also tried to quantify to which extent authors without conflicts of interest according to the disclosure list had such conflicts, in a random sample of 25% of the authors. We searched the authors’ conflicts of interest statements in scientific publications published in the three years prior to the guideline data, searched Google by combining their names with names of companies that authors of the same guideline were affiliated with, and contacted the Danish Health and Medicines Authority for additional information.

### Ethical approval

This study did not require ethical approval as it was based on publicly available information.

## Results

**Figure 1:**
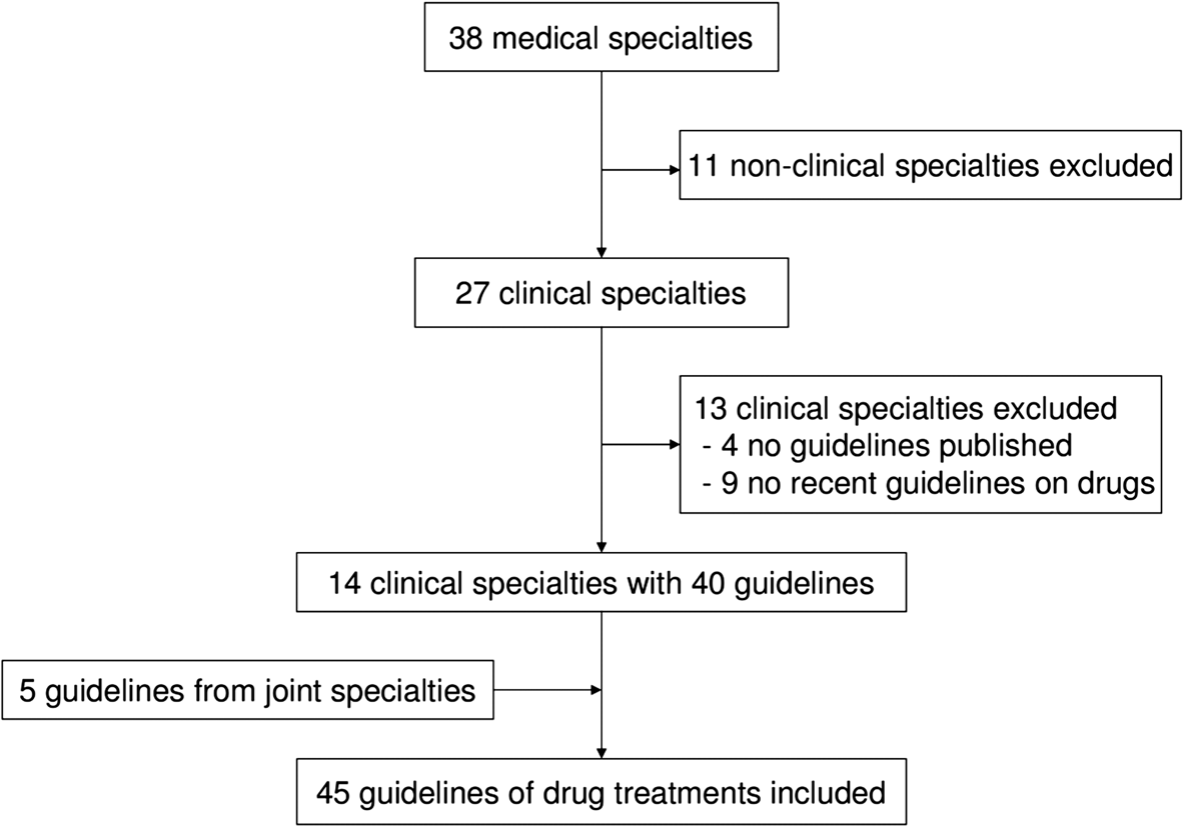
Inclusion of guidelines produced by specialty societies.

We included 45 clinical practice guidelines, 40 from 14 Danish specialty societies and 5 collaborative guidelines (Figure 1), with a total of 257 guideline authors. We excluded two authors who were psychologists and one midwife resulting in 254 physician authors. The number of authors per guideline ranged from 1 to 16 (median 5). As 7 authors participated in 2 guidelines, there were 247 unique authors. Two guidelines (4%) contained information about funding. Six drug companies supported the distribution of a guideline, but not its development. In the other guideline with information on funding, two medical societies and the Danish Institute for Rational Pharmacotherapy funded the guideline development. According to the specialty societies' replies to our emails, none of the other 43 guidelines had received funding from drug companies.

### Conflicts of interest

**Figure 2:**
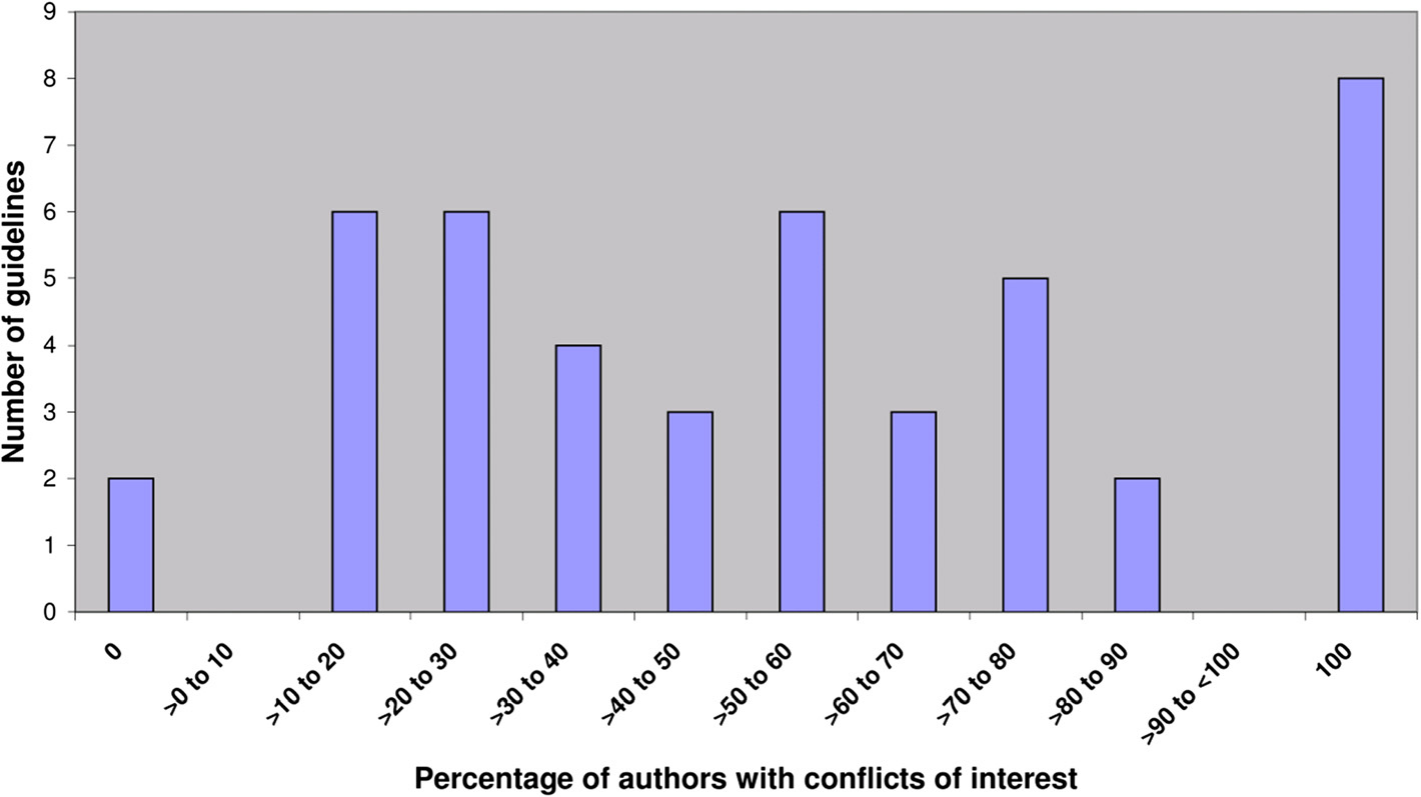
Prevalence of conflicts of interest among author groups in the 45 guidelines.

Only one guideline (2%) included a conflicts of interest statement for three of its four authors; all three were conflicted. We identified conflicts of interest for 132 additional authors, giving a total of 135 out of 254 authors (53%) with conflicts of interest. The true prevalence of conflicts of interest of guideline authors ranged from 0% to 100% among individual guidelines (Figure 2). Forty-three guidelines (96%) had one or more authors with a conflict of interest, and in only two guidelines were all authors without conflicts (4%). In 24 guidelines (53%), the majority of authors had conflicts of interest and in 8 guidelines (18%), all authors had conflicts of interest.

The most common type of conflict was consultant/advisory board member/employee followed by speaker/educational activities, investigator/research collaboration and equity/stockholder (Table 1).

**Table 1:** Types of conflicts of interest among conflicted guideline authors

	**(n = 135)**
Consultant/advisory board member/employee	83 (61%)
Speaker/educational activities	77 (57%)
Investigator/research collaboration	65 (48%)
Equity/stock	10 (7%)

The lowest prevalence of authors with conflicts was found for the Danish Society of Anaesthesiology and Intensive Care (14%), the Danish Paediatric Society (23%) and the Danish Society of Neurology (25%) (Table 2). The highest prevalence was found for the Danish Society of Dermatology (100%) and the Danish Society of Endocrinology (100%).

**Table 2:** Prevalence of authors’ conflicts of interest according to specialty society

**Name of society**	**Number of included guidelines**	**Number of authors**	**Number of authors with conflicts**
Danish Society of Anaesthesiology and Intensive Care	1	7	1 (14%)
Danish Society of Cardiology	5	22	16 (73%)
Danish Society of Child and Adolescent Psychiatry	5	19	9 (47%)
Danish Society of Dermatology	1	3	3 (100%)
Danish Society of Endocrinology	1	6	6 (100%)
Danish Society of Gastroenterology and Hepatology	5	29	9 (31%)
Danish Society of Haematology	2	17	11 (65%)
Danish Society of Infectious Diseases	1	5	4 (80%)
Danish Society of Obstetrics and Gynaecology	5	13	6 (46%)
Danish Society of Nephrology	1	8	5 (63%)
Danish Society of Neurology	1	8	2 (25%)
Danish Society of Paediatrics	5	26	6 (23%)
Danish Society of Respiratory Medicine	5	18	11 (61%)
Danish Society of Rheumatology	2	17	13 (76%)
Collaborative guidelines	5	56	33 (59%)

### Sensitivity analysis

The overall prevalence of conflicts of interest among guideline authors changed from 53% to 57% in our sensitivity analysis (simple average of percentage for each specialty society). When we searched for additional information about conflicts of interest among the 30 authors without conflicts (25% of 119) we found that 3 (10%) had conflicts that were not disclosed on the Danish Health and Medicines Authority’s list, the reason being that the activities predated our earliest available version of the list. Assuming that the 10% of authors without conflicts, according to the list, actually had conflicts, the prevalence changed from 53% to 58%.

### Guideline methodology

A description of the methods used for guideline development was found in 10 guidelines (22%). Nine of those were produced by only two societies, the Danish Society of Gastroenterology and Hepatology and the Danish Society of Obstetrics and Gynaecology, and the tenth guideline was a collaborative one. Twenty-seven guidelines (60%) included references in the text, 10 (22%) used references, but did not include them in the text, and 8 (18%) did not use references at all. Eleven guidelines (24%) graded the types of evidence; 10 of those were produced by the same two societies the Danish Society of Gastroenterology and Hepatology and the Danish Society of Obstetrics and Gynaecology and one by Danish Society of Gastroenterology and Hepatology in collaboration with five other societies.

Six out of 14 societies had instruction manuals for guideline preparation and none of those included policies on conflicts of interest.

## Discussion

We found that 53% of guideline authors had conflicts of interest, corresponding to 43 out of 45 guidelines being written by one or more authors with conflicts, and that only 2% disclosed them, in just one guideline. Most guidelines did not state how they were developed or graded the evidence, and many did not include references in the text.

Our study was based on very comprehensive data from the Danish Health and Medicines Authority, and physicians and drug companies are required by law to report their collaboration to the authority [20]. Our findings demonstrate that reliance on voluntary disclosure underestimates the prevalence of conflicts of interest substantially.

Using a publicly available disclosure list made it possible to identify undisclosed conflicts of interest, but one limitation of the list is that drug company affiliations are deleted as soon as the collaboration ends. As judged by our sensitivity analysis, this seemed to have had little impact on our results, but we suggest that such lists include affiliations up to 3 years prior to the current date, similar to the ICMJE criteria [18].

It is therefore likely that previous studies of conflicts of interest among clinical guideline authors have underestimated the prevalence of conflicts of interest, as they have relied on voluntary disclosure. We note, however, that the underreporting of conflicts of interest in our study of Danish guidelines may have been atypical, e.g. US guidelines have more disclosures [8]. We found that only six societies had instruction manuals for guideline preparation and none included policies on conflicts of interest. Thus, lack of policies on conflict of interest disclosure may have caused the extraordinary underreporting of conflicts of interest found in our study. On the other hand, this lack of transparency cannot be excused by the lack of explicit policies, as it is well known that it is important to declare conflicts of interest in biomedical publications, especially in clinical guidelines.

Albeit our study was based on Danish guidelines, our sample represents 14 different specialties and we have thereby obtained more comprehensive information on the extent of conflicts of interest among guideline authors than in other studies, which have usually only included guidelines from a few specialties [8].

In a recent systematic review by Norris and colleagues, the prevalence of conflicts of interest ranged widely among the different studies included, from 18% to 100% [8]. The large variation in prevalence may have several explanations.

Firstly, the sources used for identifying conflicts of interest may have been important. Many studies identified conflicts of interest solely based on authors’ disclosures in guidelines, which will generally underestimate the prevalence [7,21]. Other studies have identified conflicts of interest based on authors' disclosure in their additional journal publications [11,22,23] or by surveys [24,25]. However, such strategies are often inadequate [9], e.g. many journals do not include disclosure statements in their articles or have only started recently, response rates in surveys were often low, and authors often do not to disclose conflicts in their scientific publications [26,27]. A few studies have used other sources such as US patent databases [22,28].

Secondly, what constitutes a conflict of interest may also influence its prevalence. We coded a conflict of interest to be present if authors had an affiliation with a company up to 3 years prior to the published guideline. This interval differs among studies [8] as do perceptions about what constitutes a conflict of interest. For example, some studies included paid travel fees [9,21], which we did not, as such conflicts are not listed on the disclosure list. We coded any financial tie as a conflict of interest, although some ties might be related to companies producing drugs not relevant to the guideline. However, this will likely be less important, as guideline authors are usually affiliated with companies producing drugs in areas where they are experts and write guidelines in the same areas.

Thirdly, the overall extent of conflicts of interest among physicians varies between countries. In Denmark, the prevalence is approximately 8.5% [12], whereas it is 14.1% among US physicians [29].

Public disclosure lists may assist guideline issuing bodies in ensuring that all conflicts of interest are disclosed. In order for guideline end-users to judge fully the possible influence of the conflicts of interest, information on individual authors' income from drug companies is also relevant. This information will be available with the implementation of the US Sunshine act in 2014 [30], which will provide a public and comprehensive register of data about physicians’ financial relationships with the drug and device industries [15,30]. Similarly, the Danish disclosure list will in future include financial information [31]. Apart from the improved transparency in relation to guideline users, such registers may also assist researchers studying conflicts of interest.

While disclosure improves transparency, it does not remove the potential bias related to conflicts of interest [5,6,32], and a better strategy is to prohibit authors with conflicts from guideline production or minimise their influence on formulating recommendations [33]. It has been argued that authors without conflicts of interest lack the content area knowledge and skills necessary to interpret the scientific data. But this is a flawed argument. Industry relations, such as being on a company’s speakers bureau or advisory board, does not serve any academic purpose and authors can choose to avoid these relationships. Furthermore, there is a substantial pool of authors without conflicts [32]. Lastly, content area experts often have preconceptions about treatment effects, which may bias their interpretation of the evidence [34-36]. A better strategy would be to include more methodologists as guideline authors as they are often free from preconceptions and rarely have conflicts of interest.

## Conclusions

Conflicts of interest among guideline authors from Danish specialty societies were common but very rarely disclosed. Most guidelines also lacked transparency as to their development and the evidence in support of the recommendations. Thus, there is a need for better management and disclosure of conflicts of interest, and greater transparency of guideline methodology. Publicly available and law-enforced disclosure lists may assist guideline issuing bodies in ensuring that all conflicts are disclosed.

## Competing interests

The authors declare that they have no competing interests.

## Authors’ contributions

AL conceived the idea for the study. The protocol was primarily developed by AL; JBBB, JS and PCG contributed. JBBB identified guidelines and AL verified the selection. AL and JBBB extracted guideline data and JBBB and JS identified conflicts of interest. All authors participated in data analysis and writing of the paper. All authors had full access to all the data in the study. AL and JBBB are guarantors and take responsibility for the integrity of the data and the accuracy of the data analysis. All authors read and approved the final manuscript.

## Pre-publication history

The pre-publication history for this paper can be accessed here:

http://www.biomedcentral.com/1472-6939/14/19/prepub
